# Supplementary material for: Evaluation of the Nigeria national HIV rapid testing algorithm
Source: PLOS Glob Public Health. 2022 Nov 2;2(11):e0001077. doi: 10.1371/journal.pgph.0001077 (PMC10021713; doi:10.1371/journal.pgph.0001077)
Supplement: S1 Table — (DOCX) [file pgph.0001077.s001.docx]

S1 Table. Summary of the national serial HIV rapid testing algorithm compared with Geenius supplementary assay.

|  |  | **Geenius HIV-1/2 Supplementary Assay** | | |  |
| --- | --- | --- | --- | --- | --- |
|  |  | **Positive**  N (%) | **Negative**  N (%) | **Total**  N | **Agreement rate**  **% (95% CI)** |
| **National HIV Rapid Testing Algorithm (Field)** | **Positive** | 1,049 (97.4%) | 29 (27.4%) | 1,078 | 95.2  (93.2 - 97.2) |
|  | **Negative** | 28 (2.6%) | 77 (72.6%) | 105 |  |
|  | **Total** | 1,077 | 106 | 1,183 |  |
|  |  |  |  |  |  |
| **National HIV Rapid Testing Algorithm (NRL)** | **Positive** | 1,077 (100.0%) | 8 (7.5%) | 1,085 | 99.3  (97.3 - 100.0) |
|  | **Negative** | 0 (0.0%) | 98 (92.5%) | 98 |  |
|  | **Total** | 1,077 | 106 | 1,183 |  |

N = Number. CI = Confidence Interval.
