# Supplementary material for: Evaluation of the Nigeria national HIV rapid testing algorithm
Source: PLOS Glob Public Health. 2022 Nov 2;2(11):e0001077. doi: 10.1371/journal.pgph.0001077 (PMC10021713; doi:10.1371/journal.pgph.0001077)
Supplement: Attachment — Submitted filename: Response to Reviewers.docx [file pgph.0001077.s002.docx]

**Responses to the reviewer’s comments**

PGPH-D-22-00701

Evaluation of the Nigeria National HIV Rapid Testing Algorithm

PLOS Global Public Health

Dear Dr. Iriemenam,

Thank you for submitting your manuscript to PLOS Global Public Health. After careful consideration, we feel that it has merit but does not fully meet PLOS Global Public Health’s publication criteria as it currently stands. Therefore, we invite you to submit a revised version of the manuscript that addresses the points raised during the review process, in particular in positioning the testing algorithm in the context of global standards and recommendations and removing metrics such as PPV that require representative data in terms of disease prevalence to interpret appropriately.

Please submit your revised manuscript by Sep 02 2022 11:59PM. If you will need more time than this to complete your revisions, please reply to this message or contact the journal office at globalpubhealth@plos.org. When you're ready to submit your revision, log on to https://www.editorialmanager.com/pgph/ and select the 'Submissions Needing Revision' folder to locate your manuscript file.

Please include the following items when submitting your revised manuscript:

A rebuttal letter that responds to each point raised by the editor and reviewer(s). You should upload this letter as a separate file labeled 'Response to Reviewers'.

A marked-up copy of your manuscript that highlights changes made to the original version. You should upload this as a separate file labeled 'Revised Manuscript with Track Changes'.

An unmarked version of your revised paper without tracked changes. You should upload this as a separate file labeled 'Manuscript'.

Guidelines for resubmitting your figure files are available below the reviewer comments at the end of this letter.

We look forward to receiving your revised manuscript.

Kind regards,

Hannah Hogan Leslie, PhD

Academic Editor

PLOS Global Public Health

Journal Requirements:

1. Please amend your detailed Financial Disclosure statement. This is published with the article. It must therefore be completed in full sentences and contain the exact wording you wish to be published.

**Response: Thank you. This is amended.**

a. Please clarify all sources of funding (financial or material support) for your study. List the grants (with grant number) or organizations (with url) that supported your study, including funding received from your institution.

**Response: Thank you. This is done.**

b. State the initials, alongside each funding source, of each author to receive each grant.

**Response: The funding was to the implementing partner (UMB).**

c. State what role the funders took in the study. If the funders had no role in your study, please state: “The funders had no role in study design, data collection and analysis, decision to publish, or preparation of the manuscript.”

**Response: The funders had no role in study design, data collection and analysis, decision to publish, or preparation of the manuscript.**

2. We do not publish any copyright or trademark symbols that usually accompany proprietary names, eg (R), (C), or TM (e.g. next to drug or reagent names). Please remove all instances of trademark/copyright symbols throughout the text, including TM on pages.

**Response: The authors have removed all copyright and trademark symbols as suggested. Thank you.**

Additional Editor Comments (if provided):

[Note: HTML markup is below. Please do not edit.]

Reviewers' comments:

Reviewer's Responses to Questions

Comments to the Author

1. Does this manuscript meet PLOS Global Public Health’s publication criteria? Is the manuscript technically sound, and do the data support the conclusions? The manuscript must describe methodologically and ethically rigorous research with conclusions that are appropriately drawn based on the data presented.

Reviewer #1: Yes

Reviewer #2: Partly

2. Has the statistical analysis been performed appropriately and rigorously?

Reviewer #1: Yes

Reviewer #2: No

3. Have the authors made all data underlying the findings in their manuscript fully available (please refer to the Data Availability Statement at the start of the manuscript PDF file)?

The PLOS Data policy requires authors to make all data underlying the findings described in their manuscript fully available without restriction, with rare exception. The data should be provided as part of the manuscript or its supporting information, or deposited to a public repository. For example, in addition to summary statistics, the data points behind means, medians and variance measures should be available. If there are restrictions on publicly sharing data—e.g. participant privacy or use of data from a third party—those must be specified.

Reviewer #1: Yes

Reviewer #2: Yes

4. Is the manuscript presented in an intelligible fashion and written in standard English?

PLOS Global Public Health does not copyedit accepted manuscripts, so the language in submitted articles must be clear, correct, and unambiguous. Any typographical or grammatical errors should be corrected at revision, so please note any specific errors here.

Reviewer #1: Yes

Reviewer #2: Yes

5. Review Comments to the Author

Please use the space provided to explain your answers to the questions above. You may also include additional comments for the author, including concerns about dual publication, research ethics, or publication ethics. (Please upload your review as an attachment if it exceeds 20,000 characters)

Reviewer #1: This is a well written article. The investigators have done an excellent job of explaining the performance evaluation of Nigeria's national HIV rapid testing algorithm.

It was good to note that Nigeria's HIV rapid testing algorithm was performing as per expectations.

Differences in performance were noted between on-field by regular testers compared to a controlled lab environment by trained staff; the authors have provided logical reasoning for why this difference was observed and attributed it to the fact that the lab staff underwent re-orientation, re-training, and competency assessment. Those with less than 100% scores in practical evaluations were dropped from testing. The authors also provide recommendations to improve the efficiency and performance of the current algorithm. However, the manuscript cannot be accepted without a few revisions. Please find these revisions below.

• "Based on the WHO guidelines for appropriate evaluations of HIV testing technologies in Africa [10], consecutive sampling was done until 1,078 HIV positive and 1,817 HIV negative specimens were collected from all the zones". Authors need to explain the sample size calculation and justify the sample size.

**Response: Thank you. The sample size calculations and basis for calculations have been provided.**

• Figure 1- correction needed: Unigold T2 non-reactive test result is shown as N=338; correct it to 308.

**Response: Thank you very much for this observation. This is corrected.**

• Under material and methods, the first paragraph incorrectly generalizes the number of states per zone and number of sites per State selected for retrospective analysis to prospective analysis.

"Secondly, a prospective analysis of samples collected from HIV testing sites across the six geopolitical zones with varied HIV prevalence. Two States per geopolitical zone and two sites per State with an annual minimum test capacity of 1,000 HIV rapid tests per year were purposively selected for the evaluation." – this holds for retrospective analysis only. In contrast, one State per zone and three sites per State were selected in prospective analysis. The authors could provide some clarity regarding this information.

**Response: Corrections made. Thank you.**

• Retrospective study methods: Authors need to either make a reference to figure 1 under the methods section or should explain the algorithm. Authors should mention what variables were abstracted.

**Response: The authors have included figure 1 which explains the current national HIV serial rapid testing algorithm. The variables abstracted are also listed in the text.**

• Prospective study methods: Authors need to either refer to figure 1 under the methods section or should explain the algorithm. For example, it is unclear that T2 is applied to only those testing reactive to T1 unless we look at the algorithm (figure 2). Authors need to clarify it in methods to avoid confusion.

**Response: Thank you very much. The authors have added figure 1 – the national HIV serial rapid testing algorithm. The initial figure 1 in now 2 and the previous figure 2 is now 3. We referenced figure 1 in the methods as advised. We also cross referenced the National HIV testing Algorithm, which was already referenced under introduction and explained.**

•We recommend rewriting some methods sections in the prospective study section to ensure clarity.

**Response: Relevant edits made. Thank you.**

• Table 5- comparison of the National HIV Rapid Testing Algorithm performed at the Field and NRL with Genius supplementary essay- authors have provided PPV, agreement rate, and kappa statistics but not the data points. There is no way to validate this calculation. The authors have not mentioned the total number of samples re-tested with the Genius supplementary assay.

**Response: Thank you. The data points are added. The total number of samples re-tested with Geenius supplementary assay was 1,183. We have included this in this text.**

Reviewer #2: This study looked at the performance of the HIV testing algorithm in Nigeria.

Major comments

1. The relevance and relatedness of the approaches used to evaluate the algorithm in Nigeria should be reviewed in light of validation protocols that exist to do the same. If there are discrepancies, explaining why this was the case and for what purposes, advantages and disadvantages. This document should be one prioritized: https://www.who.int/publications/i/item/9789240039162. The reference (no10) provided is quite old (2002).

**Response: Thank you for the comment. As stated in the manuscript, after the 2018 NAIIS survey, the sub-analysis of Test 1 and Test 2 showed a low concordance which informed the need for the country HIV program to undertake a detailed assessment of the testing algorithm and determine if the NAIIS survey findings are consistent with the routine HIV testing in the field. This lab-based evaluation was to evaluate the current national HIV rapid testing algorithm. Our evaluation methodology has been compared with the WHO Guidelines for appropriate evaluations of HIV testing technologies in Africa in our discussion (Reference 9) and was available when the study was completed. This work was completed September 2020. The new guidance** [**https://www.who.int/publications/i/item/9789240039162 became available November 2021**](https://www.who.int/publications/i/item/9789240039162%20became%20available%20November%202021)**, more so, the referenced new WHO guidance is specifically for individual test kit verification.**

2. A major issue with this study and manuscript is that it focuses on a two-test algorithm. Since 2019, WHO has recommended countries implement a three-test algorithm, regardless of HIV prevalence rate. Presumably Nigeria is also looking to do so. Reflecting on these data in that context and how a three-test strategy may improve the performance data observed would be significantly important. Further, the algorithm presented was not aligned with WHO guidelines (from 2015) – a tie-breaker test has never been recommended, as it is presented here. Understanding the relevance then of the current algorithm presented and whether/how it can be generalized within and beyond Nigeria will be important.

**Response: Thank you very much for this important point. This evaluation was informed by the outcome of the NAIIS 2018 survey. It was to validate if the high discordance between Test 1 and Test 2 observed during the NAIIS survey was a true reflection of the test kits performance in the field, or if this is limited to the survey. The outcome of this evaluation is intended to inform decision by the FMOH on the performance of the current algorithm, and considerations for making any necessary adjustments as per WHO recommendations.**

3. Further, the patient selection in the prospective arm was purposeful rather than consecutive. Analyzing the data using PPV and NPV then is generally not possible since a purposeful selection could be biased and is not representative of existing prevalence rates (ie. ~37% with the purposeful vs 1-5% of actual prevalence in Nigeria). This should be removed and more discussion included. Further, there was a complete absence of data and discussion on the sensitivity and specificity of the algorithm in total – this is critical to understand the implications of the data and how it can be utilized in different prevalence settings (which can be calculated within a model).

**Response: The site selection for this evaluation was purposeful, however, the recruitment of study participants was consecutive sampling lab-based evaluation, and the main objective was to evaluate the current national HIV rapid testing algorithm. The low Test 1/Test2 concordance observed in the 2018 NAIIS survey fueled the decision to see if this has been existing in parts of the country and no one was privileged to notice the trend. Geenius supplementary assay which was used as the gold standard for HIV diagnosis and was not performed on HIV negatives. All HIV positives and discordants were retested using Geenius. Since all the HIV negatives were not tested using Geenius, there is no way to calculate sensitivity and specificity using Geenius as the sample size excluded the HIV negative population. However, the multiplex assay, which is another HIV confirmation assay, was used to validate the entire samples (all samples were included). Sensitivity, specificity, PPV and NPV were calculated using the multiplex assay.**

4. The variable prevalence rates for the country presented are quite confusing and unclear understanding which is correct, how they are different, why they differ, etc. More explanation and rationale here would be important. Additionally, the use of low, medium and high categories within the country seems different known considerations (ie. >5% is deemed high prevalence). Further, in Table 2 though not shown, the calculated prevalence rates were quite high in the high volume settings (higher than the suggested prevalence rates in the background). This should be further explained and likely also included in the table.

**Response: Thank you. Our study focused on evaluation of the existing national HIV testing algorithm and not on HIV prevalence calculations. This was not a survey. It was a lab-based evaluation of the national HIV testing algorithm. Our sampling methodology was not designed to establish prevalence rates; hence we did not present prevalence rates in our results. Our classification of states into low, medium, and high prevalence states is based on national prioritization for program intervention and not based on global classification of country HIV prevalence rates. We used state specific prevalence rates from the 2018 national population-based HIV impact (NAIIS) assessment for this classification.**

5. It is unclear what consent and ethical approval was sought for the retrospective study given data patient was reviewed and may have been captured.

**Response: For the retrospective component of the evaluation, we abstracted data from routine service records at the health facility level with no patient personal identifiers. In addition, the evaluation protocol (retrospective and prospective) was reviewed and approved by the country ethical review council (NHREC) and two institutional review boards (UMB and CDC).**

6. In the abstract, ‘changes’ are suggested as a rationale for the work, but were never elaborated.

**Response: Changes have been elaborated upon. Thank you.**

7. The multiplex assay used should be further described. As is, the work and testing cannot be replicated. It would be helpful to understand if this is a commercial or in-house assay, the protocol used, what type of test, etc. A brief description should be included in addition to the reference provided.

**Response: Thank you. The authors have added additional language for clarity as recommended. The details of the multiplex assay used has been published previously (Reference 12). Yufenyuy EL, Parekh BS. Development of a Multiplex Assay for Concurrent Diagnoses and Detection of HIV-1, HIV-2, and Recent HIV-1 Infection in a Single Test. AIDS Res Hum Retroviruses. 2018;34(12):1017-27. Epub 2018/07/31. doi: 10.1089/AID.2017.0279. PubMed PMID: 30056751.**

8. The first paragraph of the introduction should be rewritten, it is unclear, especially to non-HIV readers (which could be the case as this is a public health journal, not an HIV one).

**Response: Thank you for your suggestion. Edits provided for better understanding.**

9. The use of ‘testing stream’ should be reconsidered. Instead perhaps some standard language such as ‘testing site’ or ‘testing entry point’ or ‘testing location’ may be more explanatory.

**Response: A more generic term “testing point” has been used. Testing streams is a standard PEPFAR language. Thank you.**

10. In Table 2, there could be a few improvements. For example:

a. It isn’t clear what ‘standalone’ means and to what location that refers.

**Response: A footnote has been provided to explain “Standalone”. Standalone is a standard type of testing point recognized by both WHO HIV guidelines and National HIV Testing Guidelines. Thank you.**

b. It also isn’t clear what ‘ward’ means as it appears different from inpatient. Which ward?

**Response: Ward and Inpatient testing points have been merged for clarity. Thank you.**

c. There isn’t a TB setting included in this table. Is there no testing in those sites? Please explain.

**Response: TB testing points are included in “Others”. Thank you.**

11. In Table 4, it seems irrelevant to include and mention separately the performance in STI clinics given the sample size. This likely should be included in the ‘Others’ category, especially since it is likely that one or all of those five settings has a higher sample size than STI.

**Response: STI testing point has been included in “Others”, based on your recommendation. Thank you.**

12. The discussion suggests that refresher trainings will support improved field performance; however, there is no evidence and it will likely take a more comprehensive approach. This should be elaborated upon. Further, there was no data presented suggesting that staff attrition may have affected the performance in the field and thus should be removed unless evidence of impact and association can be provided.

**Response: Refresher trainings and periodic refresher trainings are required for the quality HIV testing services. Lab staff (field and NRL) were recruited and only the NRL lab staff received refresher training prior to testing. The results from the NRL staff were highly comparable with the HIV-1/2 confirmatory assays than the field lab staff results.**

**In addition, in 2015 Nigeria implemented the HIV Rapid Test for Continuous Quality Improvement (RTCQI). Many lab staff who were trained for RTCQI and posted to the selected field labs have moved on to greener pastures which could have impacted on service quality including laboratory services. We have updated the language in the discussion to show clearly that this was mainly an observation made during the evaluation. Thank you.**

13. The discussion introduces a strategy to refer all HIV positives to the laboratory for retesting. This is likely be to concerning given it will cause delays in ART initiation. This should be reflected upon more carefully.

**Response: Nigeria implements “test and start strategy” based on WHO recommendation. However, in other to maintain quality of HIV testing and diagnosis, every HIV positive is retested for verification of positives before the ART initiation. This is also consistent with WHO guidance.. This retesting is done by a certified laboratory scientist. The strategy to refer all HIV positives to the laboratory for retesting is a nationally adopted strategy. We only alluded to this strategy in our discussions. We also hypothesized based on the observed benefits of this strategy, that the benefits outweigh the demerits of delaying ART initiation. Thank you.**

Minor comments

1. On page 6 in the introduction, I believe it should be called ‘three consecutive test algorithm’ not ‘strategies’.

**Response: Thank you for your suggestion. The authors have changed it to algorithm instead of strategies.**

2. On page 12, it would be helpful to elaborate on what pediatric services means with regards to testing entry point. Is it HIV pediatric services, nutrition wards, etc.

**Response: Footnote provided to clarify what “Pediatric services” mean. Thank you.**

6. PLOS authors have the option to publish the peer review history of their article (what does this mean?). If published, this will include your full peer review and any attached files.

Do you want your identity to be public for this peer review? If you choose “no”, your identity will remain anonymous but your review may still be made public.

For information about this choice, including consent withdrawal, please see our Privacy Policy.

Reviewer #1: No

Reviewer #2: No
