# Supplementary material for: Evaluation of the Nigeria national HIV rapid testing algorithm
Source: PLOS Glob Public Health. 2022 Nov 2;2(11):e0001077. doi: 10.1371/journal.pgph.0001077 (PMC10021713; doi:10.1371/journal.pgph.0001077)
Supplement: Attachment — Submitted filename: Response to Reviewers.docx [file pgph.0001077.s003.docx]

**Response to Reviewers**

PGPH-D-22-00701R1
Evaluation of the Nigeria National HIV Rapid Testing Algorithm
PLOS Global Public Health

Journal Requirements:

Additional Editor Comments (if provided):

1) Clarity in matching the purpose of each element of the study with the reference assay used, calculations performed, and conclusions drawn. The manuscript would benefit from a clear and explicit description of how each element of the study was designed to address the study question, including the choice of quantity (e.g., PPV, NPV, kappa, etc) selected and the threshold applied for interpretation. It is evident that the results of the NAIIS prompted a re-examination of the national testing strategy, which this study then undertakes. It needs to be more clearly laid out that the retrospective assessment was undertaken to consider whether the high rates of discordance in NAIIS were also present in routine testing and whether discordance varied by location, state of the epidemic, or type of testing. The standards applied in terms of acceptable / unacceptable discordance would be helpful to spell out to aid in interpretation.

**Response: Thank you. The authors have added additional language on the reason for the retrospective component of the evaluation which was to consider if high rates of discordance in NAIIS were also present in routine testing and whether discordance varied by geopolitical zones, State, or testing points.**

The prospective evaluation requires similar description and greater clarity in the current analysis. It appears to have two purposes: 1) compare the testing algorithm when undertaken by routine testing services with the same algorithm when conducted in the national lab to see if discrepancy rate is notably higher in the field, and 2) compare algorithm performance against 2 gold standard assays. The method of sampling individuals within each selected site needs to be laid out - it is described as purposive, but based on the response to reviewer, it is only the site selection that was purposive. How were participating individuals selected - convenience in inviting everyone seeking testing on a given date range? The method of selection should be spelled out and the generalizability of participants relative to the target population of everyone being tested for HIV in Nigeria should be considered. The statistical tests used for each comparison and the standards applied should be spelled out; for the second comparison, the reader is not given details on why the Geenius assay was repeated only on positive or discordant tests while the multiplex assay was conducted on all samples. The use of both and the calculation of a range of performance statistics for each introduces confusion. The inclusion of false positive rate comparing the Geenius assay to the NRL and field testing only based on results of samples initially tested as positive or discordant is not appropriate. The use of Kappa and McNemar statistics is not well justified; kappa is used to quantify reliability and does not seem necessary when comparing a test standard to a gold standard. Overall, reducing the number of quantities calculated to ensure that those presented are clear, well justified, and interpreted in reference to a desired standard would greatly enhance the readability of the manuscript. It is also important to address the sample size calculations, which are justified based on a state-level analysis despite the fact that the main analysis is at the national level. Comparisons within each of the 6 sampled areas may be included as supplemental information, or the enrollment of individuals not required to achieve precision at a national level should be justified.

**Response: Thank you for your additional feedback. The prospective component of this evaluation study has one focus – to compare the national HIV serial testing algorithm performed at the field with the same algorithm performed at the national reference laboratory by well-trained laboratory staff. In addition, the authors included a HIV-1/2 confirmatory assay (Geenius HIV-1/2 supplementary assay) which was used as a reference for the HIV positives and discordant results. In principle, Geenius is a HIV-1/2 supplementary assay, and it is not used on HIV negatives. It is fundamentally used to confirm the true statuses of HIV positives and possibly discordant specimens based on the country’s HIV testing algorithm. As requested, the authors have removed the calculations of PPV, NPV and kappa with Geenius since the negative populations were not included with Geenius. In laboratory validations/evaluations of test kits, these measures are used – sensitivity, specificity, PPV, NPV, agreement rate and kappa statistics. Kappa statistics measures inter-rater reliability and also intra-rater reliability for qualitative (categorical) variables. We have used these with the HIV multiplex assay since all the samples were re-tested with the multiplex assay. For the prospective evaluation, participant selection was consecutive. Only those attending their HTS at the respective States and consented to participate were recruited. We removed false positive and negative from Geenius analysis as recommended.**

2) Differences between Nigeria's national approaches and WHO guidelines, including in the use of a tie breaker test and in recommending test confirmation prior to ART initiation - these issues should be made explicit and discussed in greater depth in the discussion section.

**Response: The authors have added more language to the discussion as requested. It is important to emphasize that the authors are not the Federal Ministry of Health. As stated previously, the Federal Ministry of Health requested for the evaluation and would meet to deliberate using the current WHO recommendations.**

3) Implications of the findings for NAIIS - although a stated motivation of the paper is the high discrepancy in testing during the NAIIS, this point is not returned to in the discussion. It is reassuring that the discrepancy rate is much lower in routine practice than it was in the survey, but this merits at least a mention of implications for the reliability of such an important population-based survey.

**Response:** **Thank you. The** **authors have included the testing discrepancy observed with NAIIS as suggested in the discussion. The observed higher T1/T2 discordance during NAIIS was not seen with this evaluation.**

Reviewers' comments:

Reviewer's Responses to Questions

**Comments to the Author**

1. If the authors have adequately addressed your comments raised in a previous round of review and you feel that this manuscript is now acceptable for publication, you may indicate that here to bypass the “Comments to the Author” section, enter your conflict of interest statement in the “Confidential to Editor” section, and submit your "Accept" recommendation.

Reviewer #2: (No Response)

2. Does this manuscript meet PLOS Global Public Health’s [publication criteria](https://journals.plos.org/globalpublichealth/s/criteria-for-publication)? Is the manuscript technically sound, and do the data support the conclusions? The manuscript must describe methodologically and ethically rigorous research with conclusions that are appropriately drawn based on the data presented.

Reviewer #2: Partly

3. Has the statistical analysis been performed appropriately and rigorously?

Reviewer #2: No

4. Have the authors made all data underlying the findings in their manuscript fully available (please refer to the Data Availability Statement at the start of the manuscript PDF file)?

The [PLOS Data policy](https://journals.plos.org/globalpublichealth/s/data-availability) requires authors to make all data underlying the findings described in their manuscript fully available without restriction, with rare exception. The data should be provided as part of the manuscript or its supporting information, or deposited to a public repository. For example, in addition to summary statistics, the data points behind means, medians and variance measures should be available. If there are restrictions on publicly sharing data—e.g. participant privacy or use of data from a third party—those must be specified.

Reviewer #2: Yes

5. Is the manuscript presented in an intelligible fashion and written in standard English?

PLOS Global Public Health does not copyedit accepted manuscripts, so the language in submitted articles must be clear, correct, and unambiguous. Any typographical or grammatical errors should be corrected at revision, so please note any specific errors here.

Reviewer #2: Yes

6. Review Comments to the Author

Please use the space provided to explain your answers to the questions above. You may also include additional comments for the author, including concerns about dual publication, research ethics, or publication ethics. (Please upload your review as an attachment if it exceeds 20,000 characters)

Reviewer #2: (No Response)

7. PLOS authors have the option to publish the peer review history of their article ([what does this mean?](https://journals.plos.org/globalpublichealth/s/editorial-and-peer-review-process#loc-peer-review-history)). If published, this will include your full peer review and any attached files.


**Do you want your identity to be public for this peer review?** If you choose “no”, your identity will remain anonymous but your review may still be made public.


For information about this choice, including consent withdrawal, please see our [Privacy Policy](https://www.plos.org/privacy-policy).

Reviewer #2: No
