# Supplementary material for: Evaluation of the Nigeria national HIV rapid testing algorithm
Source: PLOS Glob Public Health. 2022 Nov 2;2(11):e0001077. doi: 10.1371/journal.pgph.0001077 (PMC10021713; doi:10.1371/journal.pgph.0001077)
Supplement: Attachment — Submitted filename: Response to Reviewers.docx [file pgph.0001077.s004.docx]

**Response to Reviewers**

PGPH-D-22-00701R2
Evaluation of the Nigeria National HIV Rapid Testing Algorithm
PLOS Global Public Health

Dear Dr. Iriemenam,

Thank you for submitting your manuscript to PLOS Global Public Health. After careful consideration, we feel that it has merit but does not fully meet PLOS Global Public Health’s publication criteria as it currently stands. Therefore, we invite you to submit a revised version of the manuscript that addresses the points raised during the review process.

I appreciate the rapid response to the previous round of reviews and the changes made. As in the first revision, I believe it would be helpful if the extensive comments in the response to review were more fully reflected in the actual changes in the manuscript, particularly in making clear the purpose and interpretation of the tests conducted.

**Response: Thank you. Additional language has been added to the manuscript to aid the understanding and interpretation of the findings.**

PLOS Global Public Health is a deliberately cross-disciplinary journal with a wide readership; providing clarity on the purpose of the specific laboratory tests used is necessary to contextualize this work for that readership in a way that perhaps it would not be for a laboratory or even HIV-specific journal. In terms of statistical tests performed, certainly kappa statistics can be calculated, but the article does not interpret the added value they provide over and above the sensitivity/specificity PPV/NPV statistics laid out in testing guidelines since as WHO's. In addition, the issue raised regarding the sample size - calculated for sub-regional analysis not included in this work - remains unaddressed.

**Response: Thank you. Additional language has been added to the discussion to interpret the contributions of Agreement rates and Kappa statistics in strengthening the finding of better performance of the national HIV testing algorithm in NRL compared to field. The sampling procedure has also been further elaborated upon.**

Please submit your revised manuscript by Oct 28 2022 11:59PM. If you will need more time than this to complete your revisions, please reply to this message or contact the journal office at [globalpubhealth@plos.org](mailto:globalpubhealth@plos.org). When you're ready to submit your revision, log on to <https://www.editorialmanager.com/pgph/> and select the 'Submissions Needing Revision' folder to locate your manuscript file.

Please include the following items when submitting your revised manuscript:

- A rebuttal letter that responds to each point raised by the editor and reviewer(s). You should upload this letter as a separate file labeled 'Response to Reviewers'.
- A marked-up copy of your manuscript that highlights changes made to the original version. You should upload this as a separate file labeled 'Revised Manuscript with Track Changes'.
- An unmarked version of your revised paper without tracked changes. You should upload this as a separate file labeled 'Manuscript'.

Guidelines for resubmitting your figure files are available below the reviewer comments at the end of this letter.

We look forward to receiving your revised manuscript.

Kind regards,

Hannah Hogan Leslie, PhD
Academic Editor
PLOS Global Public Health

Journal Requirements:

Please review your reference list to ensure that it is complete and correct. If you have cited papers that have been retracted, please include the rationale for doing so in the manuscript text, or remove these references and replace them with relevant current references. Any changes to the reference list should be mentioned in the rebuttal letter that accompanies your revised manuscript. If you need to cite a retracted article, indicate the article’s retracted status in the References list and also include a citation and full reference for the retraction notice.

**Response: The authors made no changes to the reference list.**

Additional Editor Comments (if provided):


[Note: HTML markup is below. Please do not edit.]

Reviewers' comments:


[NOTE: If reviewer comments were submitted as an attachment file, they will be attached to this email and accessible via the submission site. Please log into your account, locate the manuscript record, and check for the action link "View Attachments". If this link does not appear, there are no attachment files.]

While revising your submission, please upload your figure files to the Preflight Analysis and Conversion Engine (PACE) digital diagnostic tool, <https://pacev2.apexcovantage.com/>. PACE helps ensure that figures meet PLOS requirements. To use PACE, you must first register as a user. Registration is free. Then, login and navigate to the UPLOAD tab, where you will find detailed instructions on how to use the tool. If you encounter any issues or have any questions when using PACE, please email PLOS at [figures@plos.org](mailto:figures@plos.org). Please note that Supporting Information files do not need this step.
